# A Comparative Antibacterial, Antioxidant, and Antineoplastic Potential of *Rauwolfia serpentina* (L.) Leaf Extract with Its Biologically Synthesized Gold Nanoparticles (R-AuNPs)

**DOI:** 10.3390/plants10112278

**Published:** 2021-10-24

**Authors:** Mohammad Y. Alshahrani, Zeeshan Rafi, Nadiyah M. Alabdallah, Ambreen Shoaib, Irfan Ahmad, Mohammed Asiri, Gaffar Sarwar Zaman, Shadma Wahab, Mohd Saeed, Salman Khan

**Affiliations:** 1 Department of Clinical Laboratory Sciences, College of Applied Medical Sciences, King Khalid University, P.O. Box 61413, Abha 9088, Saudi Arabia; moyahya@kku.edu.sa (M.Y.A.); irfancsmmu@gmail.com (I.A.); masiri@kku.edu.sa (M.A.); gaffarz@kku.edu.sa (G.S.Z.); 2 Nanotechnology and Nanomedicine Lab-6(IIRC), Department of Biosciences, Integral University, Lucknow 226026, India; zeddqazi@gmail.com; 3 Department of Biology, College of Science, Imam Abdulrahman Bin Faisal University, P.O. Box 1982, Dammam 31441, Saudi Arabia; nmalabdallah@iau.edu.sa; 4 Department of Clinical Pharmacy, College of Pharmacy, Jazan University, P.O. Box 114, Jazan 45142, Saudi Arabia; asahmad@jazanu.edu.sa; 5 Department of Pharmacognosy, College of Pharmacy, King Khalid University, P.O. Box 61413, Abha 9088, Saudi Arabia; shad.nnp@gmail.com; 6 Department of Biology, College of Sciences, University of Hail, P.O. Box 2440, Hail 2440, Saudi Arabia

**Keywords:** *Rauwolfia serpentina*, antibacterial, pathogenic strains, aqueous extract, cytotoxicity, R-AuNPs

## Abstract

*Rauwolfia serpentina* (*R. serpentina*), belonging to the family Apocynaceae, is a renowned medicinal herb for its different pharmacological activities such as antibacterial, antifungal, anti-inflammatory, and antiproliferative characteristics. This study has done a comparative assessment of the antibacterial, antioxidant, and anti-cancer activity of *R. serpentina* aqueous leaf extract (RSALE) with encapsulated gold nanoparticles (R-AuNPs). The R-AuNPs are prepared so that they are significant in size, monodispersed, and extremely stable. Their characterization was done by numerous parameters, including UV-visible spectroscopy (528 nm), transmission electron microscopy (~17 d. nm), dynamic light scattering (~68 d. nm), and zeta-potential (~−17 mV). Subsequently, a potent antibacterial activity was depicted via RSALE and R-AuNPs when examined by disc diffusion against various Gram-positive and Gram-negative bacterial strains. The obtained zones of inhibition of RSALE (100 mg/mL) were 34 ± 0.1, 35 ± 0.1, 28.4 ± 0.01, and 18 ± 0.01, although those of R-AuNPs (15 mg/mL) were 24.4 ± 0.12, 22 ± 0.07, 20 ± 0.16, and 17 ± 0.3 against *Staphylococcus aureus* (ATCC 25923), *Escherichia coli* (ATCC 25922), *Bacillus subtilis* (MTCC 8114), and *Streptococcus pyogenes* (ATCC 19615), respectively. However, no zone of inhibition was obtained when tested against *Proteus vulgaris* (MTCC 1771). Furthermore, the obtained MIC values for *Staphylococcus aureus* were 0.91, 0.61, and 1.15 mg/mL; for *Escherichia coli*, 0.79, 0.36, and 1.02 mg/mL; for *Bacillus subtilis* 0.42, 0.27, and 0.474 mg/mL; and for *Streptococcus pyogenes*, 7.67, 3.86, and 8.5 mg/mL of pure RSALE, R-AuNPs, and Amoxicillin (control), respectively, incorporating that R-AuNPs have been shown to have a 1.4-fold, 2.1-fold, 1.5-fold, and 1.9-fold enhanced antibacterial activity in contrast to pure RSALE tested against *Staphylococcus aureus*, *Escherichia coli*, *Bacillus subtilis*, *Streptococcus pyogenes*, and *Proteus vulgaris*, respectively. Additionally, an enhanced antioxidant potential was detected in R-AuNPs compared to RSALE evaluated by the 2,2-Diphenyl-1-Picryl Hydrazyl Radical Scavenging (DPPH) Ferric reducing antioxidant power (FRAP) assay. The determined IC 50 values of RSALE and R-AuNPs were 0.131 ± 0.05 and 0.184 ± 0.02 mg/mL, and 0.110 ± 0.1 and 0.106 ± 0.24 mg/mL via the FRAP and DPPH assays, respectively. In addition, the anti-cancer activity against the human cervical cancer (Hela) cell line was evaluated, and the MTT assay results revealed that R-AuNPs (IC_50_ 88.3 µg/mL) had an enhanced anti-cancer potential in contrast to RSALE (171.5 µg/mL). Subsequently, the findings of this study indicated that *R. serpentina* leaves and their nanoformulation can be used as a potent source for the treatment of the above-mentioned complications and can be used as a possible agent for novel target-based therapies for the management of different ailments.

## 1. Introduction

The evergreen shrub *Rauwolfia serpentina* (L.) Benth. ex Kurz., also known as Indian snakeroot, is a member of the Apocynaceae family. There are nearly 100 species of *Rauwolfia* known to exist in various climates around the world, including tropical to subtropical zones on multiple continents [1]. The stem of this upright herb is smooth, with few or no hairs. The leaf is lance-shaped, tapering with long petioles, and the fruit is spherical and purple. It is small, pink, or white, and the tuberose is prominent [2]. A very diverse range of alkaloids is present in the roots and leaf of *R. serpentina* [3]. In some cases, however, the percentage of alkaloids can vary according to where the plant is found in the world, the age of the plant, and the season of collection [4,5]. The alkaloid content of bark is over 90%, whereas root alkaloid content ranges from 1.7 to 3.0%. The active constituent of *R. serpentina* alkaloids includes ajmaline and reserpine. The other phytoconstituents are ajmalinine, reserpinine, rawolscine, serpenticine, isoajmalinerawolfinine, renoxycine rescinamine reserpilline, serpentine, sarpagine, and tetraphyllicine [4]. 

This plant possesses an extensive spectrum of pharmacological properties against many diseases, including hypertension, insomnia, diarrhea, dysentery, headache, fever, malaria, pneumonia, asthma, skin problems, and immuno-deficiency syndrome. Some practitioners use this plant to soothe patients with paranoia or schizophrenia. [6,7,8,9,10]. Nowadays, multi-drug resistant strains of bacteria are responsible for the diminished effectiveness of antibiotics, resulting in the raised spectra of untreatable bacterial infection [9]. At the same time, many experts in the medical community are still concerned about cancer therapy and the toxicity of chemotherapeutic agents. Given the urgency of finding new drugs with lower side effects, it is essential to locate them as quickly as possible. According to accepted facts, various types of herbs, including raw drugs, their phytoconstituents, and secondary constituents, are utilized in medicine as a whole, and their therapeutic potential is dependent on them [11,12]. These phytoconstituents can act as a precursor informing novel drugs and producing additive effects than the plant source.

In the science of nanomedicine and nanotechnology, gold nanoparticles have an essential role. The introduction of novel technology such as nano enhances the ability to harness nanoparticles and shows better pharmacological activity. It has also been stated that the plant extracts can reduce the inorganic salts into nanoparticles and stabilize them by capping them [13,14]. They are highly effective as antibacterial and anti-inflammatory agents, and they are extensively employed [15]. They enhance the efficacy and affinity of the drug and thus can manage various ailments [16,17].

In light of the explanations mentioned above, this study was designed to synthesize and characterize the R-AuNPs. Further, a comparative research was carried out to analyze the enhanced antibacterial, antioxidant, and anti-cancer potential of *R. serpentina* aqueous extract of the leaf (RSALE) and R-AuNPs.

## 2. Materials and Methods

### 2.1. Plant Collection and Extract Preparation

Fresh plants of Rauwolfia serpentina were obtained from the herbarium garden Integral University Campus, Lucknow, India, in May 2021. The plant specimen was authenticated by the taxonomist at the Department of Pharmacognosy, Faculty of Pharmacy, Integral University, Lucknow, U.P., India, with voucher specimen reference no. (IU/PHAR/HRB/21/05). The new leaf of the plant was collected, rinsed repeatedly with distilled water to remove any impurities from their surfaces. Then, leaves were cut into small pieces and air-dried, grounded with double distilled water using pestle mortar placed in a tray filled with ice to avoid protein denaturation. The aqueous extract was prepared by adding 50 g of air-dried powder with 100 mL of double-distilled water. Afterward, the mixture was left to dry at room temperature. The stock concentration of 500 mg/mL of the solution mixture was prepared and stored at 4 °C for future experiments [18].

### 2.2. R. serpentina Mediated Synthesis of Gold Nanoparticles (R-AuNPs)

In-vitro synthesis of AuNPs was performed by incubating 1 mM Chloroauric acid (HAuCl_4_) prepared in phosphate buffer (50 mM, pH 7.4) and RSALE at a ratio of 1:1 (*v*/*v*) to a volume of 30 mL. The reaction was further incubated at 40 °C for 48 h. Another reaction mixture containing only RSALE was used as a control [18]. After a definite period, the sample was ejected and analyzed on a Biospectrum-Kinetics spectrophotometer (Eppendorf) using a quartz cuvette having the path length of 1 cm to affirm the synthesis of *R. serpentina* encapsulated gold nanoparticles (R-AuNPs). Subsequently, the solution was filtered using a syringe with a filter having a pore size of 2 µm, and the unbound proteins were expelled using 50% *v*/*v* ethanol treatment followed by centrifugation (30,000× *g*) for 30 min. Finally, the samples were washed twice by Milli Q water and utilized for further characterization.

### 2.3. Characterization of R-AuNPs

The transformation of gold salts into gold nanoparticles was investigated using the Shimadzu UV-1601 dual-beam spectrophotometer. This measurement has a spatial resolution of one nanometer (200 nm to 800 nm). The technique is based on reducing metal salts to synthesized AuNPs results in a color change. A single drop of an AuNPs suspension was applied to carbon-coated copper TEM grids. It was then analyzed using a Hillsboro, Oregon-based Tecnai G2 Spirit transmission electron microscope equipped with a BioTwin lens configuration. An 80 kV80-kV accelerating voltage powers the system. Subsequently, dynamic light scattering (DLS) was used to determine the hydrodynamic radius of the nanoparticles produced. Additionally, a Zetasizer Nano-ZS (ZEN3600 Malvern Instrument Ltd., Malvern, United Kingdom) was used to determine the particle size and zeta potential. The size and shape of gold nanoparticles were also determined using transmission electron microscopy.

### 2.4. Determination of Antibacterial Activity by Disc Diffusion Method

The disc diffusion method [19] was used to determine the antibacterial properties of RSALE and R-AuNPs. For antibacterial assay analysis, pure cultures of *Staphylococcus aureus* (ATCC 25923), *Escherichia coli* (ATCC 25922), and *Streptococcus pyogenes* (ATCC 19615) were obtained from American Type Culture Collection. However, *Bacillus subtilis* (MTCC 8114) and *Proteus vulgaris* (MTCC 1771) were obtained from the Microbial Type Culture Collection and Gene Bank (MTCC), housed at the Institute of Microbial Technology (IMTECH), Chandigarh, India. The suspensions of the strains tested were standardized to 0.5 McFarland. The suspension was obtained from overnight trypticase soy broth (TSB) cultures and placed on the surface of Mueller–Hinton (M.H.) agar for the study, as mentioned earlier. During the experiment, 50 µL of various concentrations of R-AuNPs (10, 20, 40, 60 and 80 mg/mL) and various concentrations of crude RSALE (10, 25, 50, 75, and 100 mg/mL), negative control (PBS) and positive control amoxicillin (25 mg/mL) were added to the wells of MH agar plates. The experiments were conducted in triplicate, and the agar plates were incubated overnight at 37 °C. Following that, the diameter of the inhibitory zone was determined. The experiment was conducted in triplicates in the same experimental conditions.

### 2.5. Minimal Inhibitory Concentration (MIC) Determination

To quantify the antibacterial capabilities and determine the MIC of the pure RSALE, R-AuNPs, and Amoxicillin (used as control), the test was performed on a sterile 96-well plate, as described in many studies [20,21]. The crude RSALE and R-AuNPs were serially diluted in nourishing broth. Then, each well of 96-well plates was incubated for 24 h at 37 °C with a standardized suspension of bacteria (10^3^ cells/mL). After 24 h, an ELISA reader [(Microplate Reader (BIORAD-680)] was used to read the plate at 625 nm, and subsequently, the reading was recorded for further use.

The results of the antibacterial study and MIC values indicated that *R. serpentina’s* efficacy is significantly increased due to the encapsulation over the AuNPs. At considerably lower doses, it was observed that R-AuNPs hindered the growth of a variety of Gram-negative and Gram-positive bacterial strains. There may be a synergy between the antibacterial characteristics of the AuNPs and the *R. serpentina*, which may explain why the *R. serpentina’s* antibacterial activity enhanced after encapsulation over AuNPs. AuNPs exert their antibacterial effects primarily through two mechanisms: collapsing the membrane potential, which inhibits ATPase activity and hence the ATP level; the other is by interfering with the ribosome’s component binding to tRNA. However, AuNPs do not have ROS-related pathways, which are used by many bactericidal drugs and nanomaterials.

### 2.6. Antioxidant Assay

The 2,2-diphenyl-1-picrylhydrazyl (DPPH) and ferric reducing ability of plasma or plants (FRAP) assays were used to determine the number of antioxidants in the RSALE and R-AuNPs. At various concentrations (0.0625–1 mg/mL), the RSALE and R-AuNPs were dissolved in methanol. L-ascorbic acid and Trolox were used as a standard for the study, while methanol was employed as a negative control. The experiment was performed on a Biospectrum-Kinetics spectrophotometer (Eppendorf in triplicates and mean values were recorded.

#### 2.6.1. 2,2-Diphenyl-1-Picryl Hydrazyl (DPPH) Radical Scavenging Assay

The extract and R-AuNPs were prepared for the experiment at various concentrations (0.05 to 1 mg/mL). A solution of DPPH (0.1 mM) in ethanol was prepared, and 1.0 mL of this solution was added to 3.0 mL of all the extracts solution in water at different concentrations. This extraction solution was then mixed with 100 mM of DPPH and incubated at 37 °C in the dark. Then, the sample was incubated, and the absorption was recorded at 490 nm on a Biospectrum-Kinetics spectrophotometer (Eppendorf) [22]. L-ascorbic acid was used as a control. To estimate the level of scavenging activity, we used the following formulae:

Scavenging activity (%) = [(Absorbance of control − Absorbance of extract)/(Absorbance of control)] × 100

#### 2.6.2. Ferric Reducing Antioxidant Power (FRAP) Assay

The FRAP assay was used to measure the antioxidant potential of RSALE and R-AuNPs spectrophotometrically [23]. The FRAP indicator was made by taking 300 mM acetate buffer, 10 mL TPTZ (2,4,6-tripyridyl-s-triazine) in 40 mM HCl, and 20 mM FeCl_3_·6H_2_O in the ratio of 10:1:1 at ambient temperature. The working solution of FRAP was freshly prepared, and 5 µL of plant extract (in various concentrations ranging from 0.05 to 1 mg/mL) and R-AuNPs solution were mixed, then placed in a 37 °C incubator for exactly 1.5 h. Subsequently, the reduction of Fe^2+^ ions to Fe^3+^ ions was confirmed by the appearance of intense blue color. Afterward, on a Biospectrum-Kinetics spectrophotometer (Eppendorf), the absorbance was recorded at 593 nm and compared with a blank reagent that includes a 4 mL FRAP reagent with 5 µL of distilled water. The calibration curve was plotted to determine the FRAP values. A graph was developed with ferrous sulfate concentrations versus concentrations of Trolox as standard. Finally, the FRAP values were determined by comparing the absorbance in the test sample at increasing concentration of ferric ion, which was stated as Trolox equivalent/gram of sample.

### 2.7. Cytotoxicity Analysis against Human Cervical Cancer Cell Hela by MTT Cell Proliferation Assay

A cell proliferation assay was used to estimate the cytotoxic potential using MTT (3-(4,5-dimethylthiazol-2-yl)-2,5-diphenyltetrazolium bromide) of the RSALE and R-AuNPs. The formation of the purple formazan compound generated in viable cells was determined calorimetrically [24,25]. The cytotoxicity test was performed against the human cervical cancer cell line Hela. Counting and plating were performed in 96-well plates (1 × 10^4^ cells per well) aseptically, and incubation was done at 37 °C for 24 h [26]. Furthermore, during the analysis procedure, the monolayer of cells after treatment with crude RSALE and R-AuNPs was further incubated at 37 °C for another 24 h in a 5% CO_2_ incubator. Subsequently, the media was removed from the wells, and cells were stained with 50 µL of (3-(4,5-dimethylthiazol-2-yl)-2,5-diphenyltetrazolium bromide) MTT stain (5 mg/mL in PBS). The plates were then incubated for another 4 h in a 5% CO_2_ incubator. Then, the plates were incubated for another 4 h in a 5% CO_2_ incubator. Later, 150 µL of DMSO (Dimethyl sulfoxide) was added to each well to dissolve the formazan complex formed by surviving cells that form purple. Subsequently, the absorbance at 570 nm was recorded using ELISA reader ((Microplate Reader (BIORAD-680)). The results were obtained by calculating percentage inhibition values. The experiment was performed in triplicate, and mean values were determined as IC_50_ 196 µg/mL.

### 2.8. Data Analysis

All the samples were evaluated in triplicate for each assay, and the findings are reported as mean ± S.D. The one-way analysis of variance (ANOVA) was used to assess the results using GraphPad Prism version 4.02 for Windows (Graph Pad Software, San Di-ego, CA, USA). The significance level for each experiment is represented in the respective figures and tables of this manuscript [27,28].

## 3. Results and Discussion

### 3.1. Synthesis of Rauwolfia serpentina Extract Mediated Gold Nanoparticles

This study used RSALE as a reducing and stabilizing agent, whereas 1 mM Chloroauric acid (HAuCl_4_) served as the gold precursor. It was hypothesized that the aqueous extract induces the formation of R-AuNPs through the action of its reducing enzymes and capping agents such as secondary metabolites, which combine to reduce AuCl4 (+3 oxidation state) to Au (0 oxidation state). The reduction of gold was confirmed visually by the change in color of the extract from green to ruby red, confirming the formation of R-AuNPs [29]. 

### 3.2. Characterization of R-AuNPs

**Figure 1:**
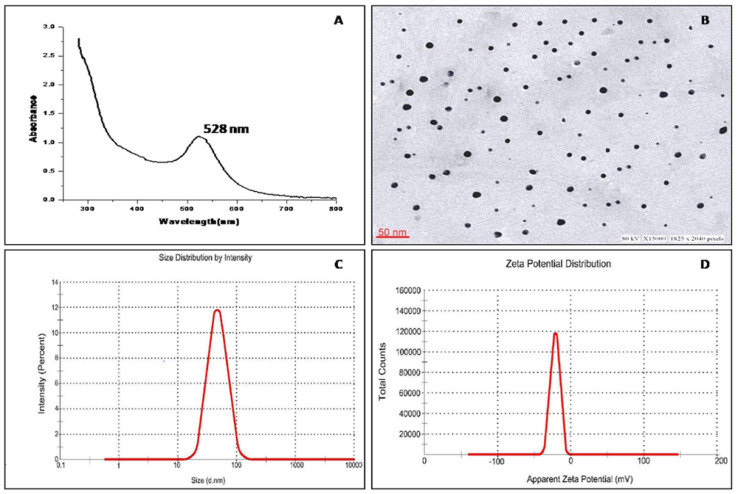
The figure shows results of diverse characterization characteristics of *Rauwolfia serpentina* extract mediated gold nanoparticles (R-AuNPs). (**A**) U.V. visible spectroscopy shows absorption, (**B**) the transmission electron microscope illustrations of the precise size, shape, and 2-dimensional morphology of R-AuNPs, (**C**) the dynamic light scattering technique shows average particle size and profile of the particle size distribution of R-AuNPs, and (**D**) the Zeta potential displays the colloidal stability of nanoparticles (R-AuNPs).

In noble metal nanoparticles, an unfamiliar phenomenon is observed due to surface plasmon resonance (SPR). This imparts the quality of intense electromagnetic fields onto the nanoparticle’s surface, resulting in scattering and absorption [17]. Thus, the formation of R-AuNPs was confirmed herein using U.V. vis spectra (Figure 1A). The absorption peak was observed at 528 nm, which corresponds to the SPR band of the R-AuNPs. The phytoconstituents in RSALE reduced the gold salt (HAuCl4) into AuNPs and encapsulated the AuNPs, preventing the nanoparticles from aggregating and providing stability to the R-AuNPs. The change in color from light yellow to ruby red indicated the successful synthesis of R-AuNPs, and the result of the SPR band confirmed that at 528 nm.

However, there was no discernible peak for RSALE. The transmission electron microscope (TEM) was used to determine the precise size, shape, and 2-dimensional morphology of R-AuNPs, which was determined to be 17 d. nm with spherical shape and monodispersed parameter (Figure 1B). The technique of dynamic light scattering (DLS) was used to determine the average particle size and profile of the particle size distribution of R-AuNPs. R-AuNPs had an average particle size of 68 d. nm and a polydispersity index (PDI) of 0.215, indicating a homogeneous size distribution, as shown in Figure 1C.

The zeta potential of R-AuNPs was also investigated (Figure 1D). Generally, a zeta value of −20 mV is needed for the colloidal stability of nanoparticles [30]. The zeta potential of the prepared R-AuNPs was −17 mV, indicating the high strength of the particles. When the aqueous dispersion of R-AuNPs was observed at room temperature, no clumping or accumulation was observed. This was most likely due to the electrostatic repulsive forces of the gold nanoparticles. This repulsion prevents the nanoparticles from coming into contact with one another.

### 3.3. Antibacterial Activity

There are different microbial pathogens known for causing several infectious diseases. These pathogens have developed mutation and resistance to several antibiotics due to inappropriate use. Hence, several researchers are finding alternative systems of medicine for the treatment. The present study analyzed the antibacterial potential of crude *Rauwolfia serpentina* aqueous extract and R-AuNPs against Gram-negative (*Escherichia coli*, and *Proteus vulgaris)* and Gram-positive (*Staphylococcus aureus*, *Streptococcus pyogenes*, and *Bacillus subtilis)* bacterial strains by the disc diffusion method. The *Rauwolfia serpentina* aqueous extract and R-AuNPs showed potent antibacterial activity against all the aforementioned bacterial strains except *P. vulgaris*. 

The maximum activity against *S. aureus*, *E. coli*, *B. subtilis*, and *S. pyogenes* was observed at a concentration of 100 mg/mL crude aqueous extract of *R. serpentina*, with inhibitory zones of 34 ± 0.1, 35 ± 0.1, 28.4 ± 0.01, and 18 ± 0.01, respectively (Table 1). However, no zone of inhibition was observed in the analysis against *Proteus vulgaris.* Additionally, in the case of positive control amoxicillin (25 mg/mL), the obtained zone of inhibition was 8 ± 0.02 **, 7 ± 0.5 **, 6 ± 0.3 **, 11 ± 0.02 *** against *S. aureus*, *E. coli*, *B. subtilis*, and *S. pyogenes,* respectively, with no zone of inhibition observed against *Proteus vulgaris.*

However, R-AuNPs have shown a significantly high zone of inhibition at a poor concentration (15 mg/mL) comparative to the crude RSALE and Amoxicillin. R-AuNPs established the zone of inhibition of 24.4 ± 0.12, 22 ± 0.07, 20 ± 0.16, and 17 ± 0.3 against *S. aureus, E. coli*, *Bacillus subtilis*, and *Streptococcus pyogenes* Table 2). However, no zone of inhibition was observed against *Proteus vulgaris*. During the experiment, Dimethyl sulfoxide (DMSO) was used as a negative control. The enhanced antibacterial activity observed against the aforesaid bacterial strains might be due to the synergistic effect of the gold nanoparticles and active phytoconstituents of the *R. serpentina*, which are present over the surface of the gold nanoparticle that resulted from the synthesis process. 

### 3.4. Determination of Minimal Inhibitory Concentration of RSALE and R-AuNPs

**Figure 2:**
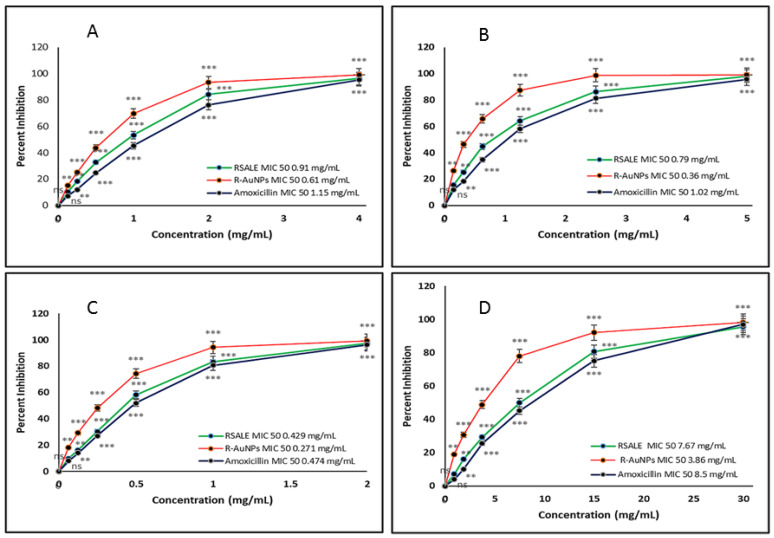
Determination of Minimum Inhibitory Concentration (MIC) of pure RSALE, R-AuNPs, and amoxicillin (positive control) against (**A**) *Staphylococcus aureus* (**B**) *Escherichia coli* (**C**) *Bacillus subtilis*, and (**D**) *Streptococcus pyogenes*. The experiment was repeated in triplicate, and the data shown are the means ± S.D. mean (*n* = 3). Significantly different from control at *** *p* < 0.001, Significantly different from control at ** *p* < 0.01, Non-significantly different from control at ^ns^ *p* > 0.05.

The MIC is the lowest concentration of RSALE and AuNPs that completely inhibits bacterial growth, and MIC_50_ is the concentration of RSALE and R-AuNPs that inhibits 50% of the bacterial population. The MIC_50_ of RSALE and R-AuNPs against several Gram-negative and Gram-positive bacterial strains were recorded. However, Amoxicillin was used as a standard antibiotic during the experiment. The quantified MIC_50_ values were *Staphylococcus aureus*, 0.91 mg/mL, 0.61 mg/mL, and 1.15 mg/mL (Figure 2A); *Escherichia coli*, 0.79 mg/mL, 0.36 mg/mL, and 1.02 mg/mL (Figure 2B); *Bacillus subtilis*, 0.42 mg/mL, 0.27 mg/mL, and 0.474 mg/mL (Figure 2C); *Streptococcus pyogenes*, 7.67 mg/mL, 3.86 mg/mL, and 8.5 mg/mL (Figure 2D) of pure RSALE, R-AuNPs, and Amoxicillin (control), respectively. The findings, as mentioned earlier, indicated that R-AuNPs had a 1.4-fold, 2.1-fold, 1.5-fold, and 1.9-fold increased antibacterial potential compared to pure RSALE when tested against *Staphylococcus aureus*, *Escherichia coli, Bacillus subtilis*, and *Streptococcus pyogenes,* respectively. However, compared to the control (Amoxicillin), R-AuNPs showed an enhanced antibacterial potential of 1.8 folds, 2.8 folds, 1.7 folds, and 2.2 folds against *Staphylococcus aureus*, *Escherichia coli*, *Bacillus subtilis*, and *Streptococcus pyogenes*, respectively. The potent antibacterial activity observed is might be due to the presence of phytoconstituents present in the plant. Recent studies reported that plant rich in alkaloids resembles the significant amount of antibacterial activity. Additionally, our extracts also contain flavonoids, tannins, saponins, gums, and mucilages in a significant amount which might be the reason for its extraordinary antibacterial potential [31]. A study by Sarika et al. in the year 2012 reported the presence of four different indole alkaloids like ajmalicine, ajmaline, yohimbine, and reserpine in *R. serpentine* [32,33]. 

Our MIC results suggest that the R-AuNPs are much more effective at lower doses than pure RSALE and Amoxicillin used against the aforesaid bacterial strains. The encapsulation of the R-AuNPs with active phytoconstituents present in *R. serpentina* resulted in the decreased quantity of the *R. serpentina* and the increased efficacy and stability of R-AuNPs. As the AuNPs are acting as a carrier for the *R. serpentina*, they (AuNPs) are also exerting the antibacterial potential by collapsing the membrane potential, hindering the ATPase activities and resulting in a change in the ATP level of the bacterial cell wall [34]. The combined antibacterial capabilities of the *R. serpentina* and the AuNPs could boost the antibacterial effectiveness of the R-AuNPs.

Previous studies have demonstrated similar findings of antibacterial activity in aqueous and methanolic extracts of *R. serpentina* root, examined against *Enterococcus faecalis, Micrococcus luteus, Staphylococcus aureus*, and *Streptococcus pneumoniae* by the agar well diffusion method [21]. 

### 3.5. Antioxidant Activity Analysis

**Figure 3:**
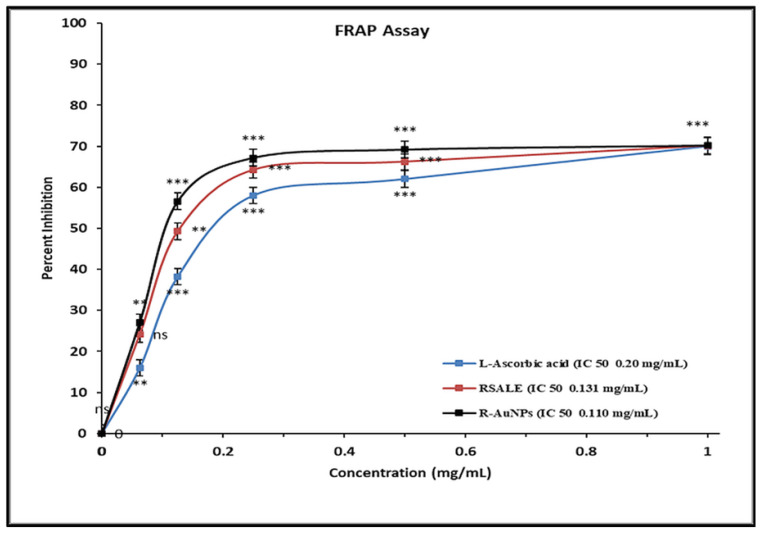
The scavenging effect of RSALE, R-AuNPs, and L-ascorbic acid (standard) by FRAP Assay. The different concentrations of extracts used were 0.05 to 1 mg/mL. The data represent the percentage inhibition values and are expressed as mean ± S.D. (*n* = 3). Significantly different from control at *** *p* < 0.001, Significantly different from control at ** *p* < 0.01, Non-significantly different from control at ^ns^ *p* > 0.05.

RSALE and R-AuNPs exhibited high antioxidant activity and a high capacity for scavenging free radicals. The FRAP assay portrayed the IC_50_ values of RSALE and R-AuNPs to be 0.131 ± 0.05 and 0.110 ± 0.1 mg/mL, respectively. However, the L-Ascorbic acid (standard) portrayed an IC_50_ value of 0.20 ± 0.2 mg/mL, as shown in Figure 3.

**Figure 4:**
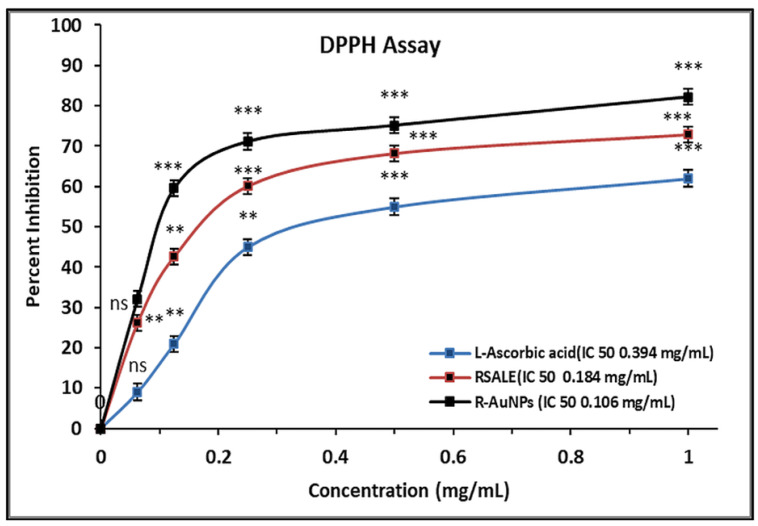
The scavenging effect of RSALE, R-AuNPs, and L-ascorbic acid (standard) by the DPPH method. The different concentrations of extracts used were 0.05 to 1 mg/mL. The data represent the percentage of DPPH inhibition, and values are expressed as mean ± S.D. (*n* = 3). Significantly different from control at *** *p* < 0.001, Significantly different from control at ** *p* < 0.01, non-significantly different from control at ^ns^ *p* > 0.05.

The DPPH assay represented IC_50_ values of RSALE and R-AuNPs to be 0.184 ± 0.02 and 0.106 ± 0.24 mg/mL, respectively. However, the L-Ascorbic acid (standard) portrayed an IC_50_ value of 0.394 ± 0.1 mg/mL, as shown in Figure 4. Similar findings are obtained in a 2015 study according to which *R. serpentina* exhibited significant antioxidant activity verified by the DPPH method, with flavonoids being identified as the active ingredient [35]. 

### 3.6. Cytotoxicity by MTT Cell Proliferation Assay

**Figure 5:**
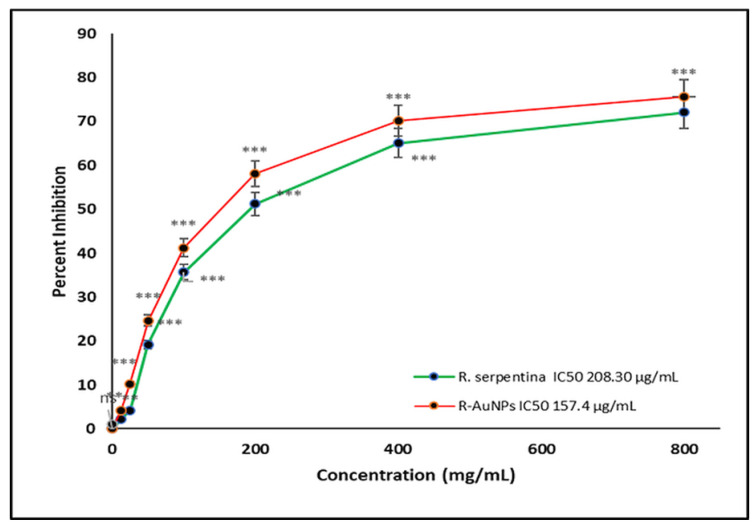
Comparative IC_50_ values of RSALE and R-AuNPs by MTT cell proliferation assay tested against normal human keratinocyte HaCat cells. The different concentrations of extracts and R-AuNPs used ranged from 12.5 to 800 µg/mL. The data represent the percentage inhibition values and are expressed as mean ± S.D. (*n* = 3). Significantly different from control at *** *p* < 0.001, Significantly different from control at ** *p* < 0.01, Non-significantly different from control at ^ns^ *p* > 0.05.

**Figure 6:**
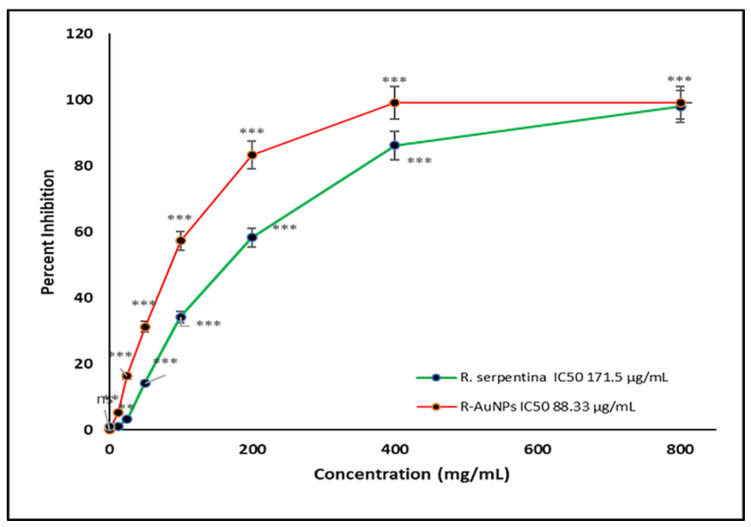
Comparative IC_50_ values of RSALE and R-AuNPs by MTT cell proliferation assay tested against *human cervical cancer cell line, Hela*. The different concentrations of extracts and R-AuNPs used ranged from 12.5 to 800 µg/mL. The data represent the percentage inhibition values and are expressed as mean ± S.D. (*n* = 3). Significantly different from control at *** *p* < 0.001, Significantly different from control at ** *p* < 0.01, Non-significantly different from control at ^ns^ *p* > 0.05.

**Figure 7:**
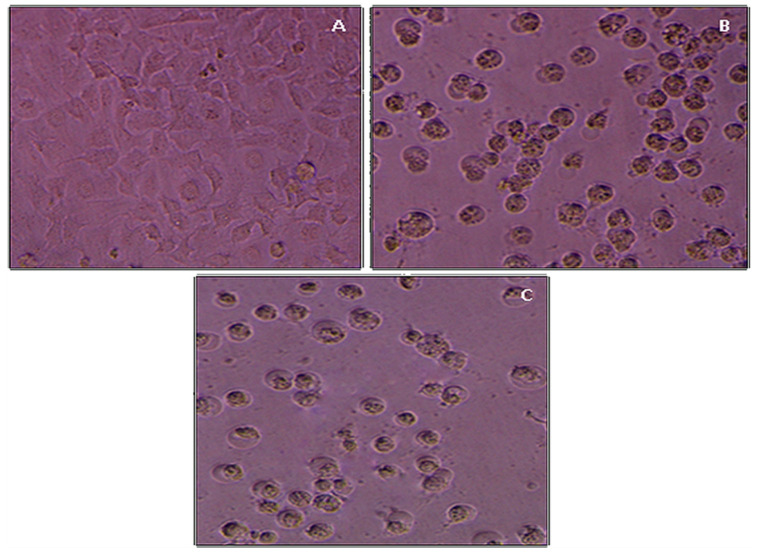
Anti-cancer effects of RSALE and R-AuNPs against Hela cells evaluated by MTT assay: (**A**) Photomicrograph of the control group, (**B**) RSALE treated Hela cells, and (**C**) R-AuNPs treated Hela cells at obtained I.C. 50 concentrations.

The MTT cell propagation assay was used to determine the cytotoxicity analysis of *R. serpentina* extract and R-AuNPs against normal human keratinocyte HaCat cells. The anti-cancer potential of the RSALE and R-AuNPs against cancerous cell lines (Hela). Our cytotoxicity analysis results over HaCat cells demonstrated that RSALE and R-AuNPs have an IC_50_ value of 208.30 and 157.7 µg/mL against HaCat cells (Figure 5). However, when the samples above were investigated for anti-cancer potential, it was found that the RSALE and R-AuNPs inhibited Hela (cervical carcinoma) by 50% at 171.5 µg/mL and 81.3 µg/mL, respectively (Figure 6). Microscopically, significant morphological changes were observed in treated cancer cell lines compared to controls. Hela cells treated with RSALE and R-AuNPs demonstrated a cytotoxic effect, as indicated by cellular morphology changes. The morphological changes include cytoplasmic condensation and cell clumping, as well as growth inhibition (Figure 6). The cytotoxic effects on Hela cells were dose-dependent and resulted in cell death and senescence. Cell death and AuNPs-induced apoptosis are two morphological changes observed in Hela (Figure 7). Our results also portrayed that RSALE and R-AuNPs are cytotoxic to cancerous Hela cells at much lower concentrations than normal human keratinocyte HaCat cells. This can be justifying the IC_50_ doses of the RSALE, and R-AuNPs obtained during the anti-cancer potential analysis are safer than the normal cells. 

Similarly, an ethanolic leaf extract of *R. serpentina* was found to have antiproliferative activity against the cancerous cell line Hela, with an IC_50_ value of 196 µg/mL [3] Similarly, in a recent study [36], the hexane extract of *Rauvolfia tetraphylla* demonstrated cytotoxicity against a variety of cancer cell lines, including cervical (HeLa), lung (A549), colon (Caco 2), and breast (MCF7 and MDA MB 231).

Additionally, anti-cancer analysis of pure RSALE and R-AuNPs against the human cervical cancer cell line, Hela, demonstrated that both *R. serpentina* and R-AuNPs are cytotoxic to the cancer cells. Nonetheless, R-AuNPs are significantly more effective than RSALE at low concentrations. The same results are observed in our studies; that indicated that the stable gold bioconjugates (AuNPs coupled with drug) choose endocytosis as a mode of entry into the cells and are known to perform far better than pure medicines at a considerably lower concentration, with significantly fewer side effects, which makes targeted drug delivery to cancerous cells a very appealing use of nanotechnology [37,38]. However, the ability of the R-AuNP conjugates to enter the cell may depend on the physical and interfacial properties of the N.P.s, such as surface charge, size, shape, and hydrophobicity. Additionally, it is dependent on the cell type and plasma membrane characteristics [39], such as receptor density, receptor type, receptor recycling rate, and membrane fluidity [40]. They can then deliver the drug straight into the cancer cells’ cytoplasm, followed by their entrance into the nucleus.

Additionally, the natural anti-cancer products can cause cell death by various mechanisms, including cell cycle regulation and activation of apoptotic and nonapoptotic pathways (such as autophagy, necrosis, mitotic catastrophe, and senescence). The apoptotic pathway has received more interest in research in the case of plant-derived compounds. During cell development, programmed death is characterized by distinct morphological and biochemical changes. The morphological alterations include decreased cell size, increased cytoplasmic density, organelle compression, and chromatin condensation [41].

On the other hand, biochemical alterations include protein cleavage, cross-linking of proteins, DNA fragmentation, mitochondrial fragmentation, and phagocytic recognition. The intrinsic (mitochondrial) and extrinsic (death receptor) apoptotic pathways involved overlap and interact. The p53 and lysosomal routes are two possible sub-pathways [42]. Therefore, R-AuNPs show a potent anti-cancer effect aiding its entry into the cancer cell and exerting the anti-cancer activities synergistically with the couples of *R. serpentina* molecules.

## 4. Conclusions

The current study demonstrates that the aqueous leaf extract of *R. serpentina* has less stability than R-AuNPs. The size of a chemically synthesized gold nanoparticle (R-AuNPs) was 17 d. nm and we conclude that our preparation is monodispersed, spherical in morphology, and highly stable (zeta potential ~ −17 mV). Our FRAP and DPPH assay results also indicated that when *R. serpentina* is coupled with gold nanoparticles, its antioxidant potential and free radical scavenging activity are enhanced (R-AuNPs). In conclusion, we can say that R-AuNPs (offered at a lower dose than pure *R. serpentina*) can substitute for *R. serpentina* due to the increased antibacterial, antioxidant, and anti-cancer potential. Thus, the study’s findings indicate that the crude *R. serpentina* aqueous leaf extract and R-AuNPs may effectively treat bacterial infections and act as an anti-cancer agent. However, additional investigations are required to determine the active phytoconstituent of the *R. serpentina,* which might be responsible for reducing gold salt into its nano form and coupling on its surface to stabilize the synthesized R-AuNPs. Additionally, more research is also needed to determine the in vivo activity of R-AuNPs before they can be declared suitable drug carriers in the medical domain.

## Figures and Tables

**Table 1:** Antibacterial activity of RSALE and Amoxicillin (used as positive control) against Gram-positive and Gram-negative bacteria.

Conc. of Sample	Zone of Inhibition (mm)									
	*Staphylococcus aureus*		*Escherichia coli*		*Bacillus subtilis*		*Streptococcus pyogenes*		*Proteus vulgaris*	
	*R. serpentina* Leaf Extract	Amoxicillin (Positive Control)	*R. serpentina* Leaf Extract	Amoxicillin (Positive Control)	*R. serpentina* Leaf Extract	Amoxicillin (Positive Control)	*R. serpentina* Leaf Extract	Amoxicillin (Positive Control)	*R. serpentina* Leaf Extract	Amoxicillin (Positive Control)
**Negative Control**	0	-	0	-	0	-	0	-	0	-
**10 mg/mL**	9 ± 0.5 ***	-	10 ± 0.05 ***	-	8 ± 0.02 **	-	N.Z. ^ns^	-	N.Z. ^ns^	-
**25 mg/mL**	13 ± 0.05 ***	8 ± 0.02 **	12 ± 0.07 ***	7 ± 0.5 **	10 ± 0.01 ***	6 ± 0.3 **	NZ ^ns^	11 ± 0.02 ***	NZ ^ns^	NZ ^ns^
**50 mg/mL**	25.4 ± 0.3 ***	-	20 ± 0.5 ***	-	18 ± 0.2 ***	-	N.Z. ^ns^	-	N.Z. ^ns^	-
**75 mg/mL**	32.3 ± 0.4 ***	-	30 ± 0.6 ***	-	19 ± 0.05 ***	-	13 ± 0.05 ***	-	N.Z. ^ns^	-
**100 mg/mL**	34 ± 0.1 ***	-	35 ± 0.1 ***	-	28.4 ± 0.01 ***	-	18 ± 0.01 ***	-	N.Z. ^ns^	-

Significantly different from control at *** *p* < 0.001, significantly different from control at ** *p* < 0.01, non-significantly different from control at ^ns^ *p* > 0.05. Note: N.Z.—No zone of inhibition; the values are mean ± standard deviation (*n* = 3).

**Table 2:** Antibacterial activity of *R-AuNPs* against Gram-positive and Gram-negative bacterial strains.

Conc. Of R-AuNPs	Zone of Inhibition (mm)				
	*Staphylococcus aureus*	*Escherichia coli*	*Bacillus subtilis*	*Proteus vulgaris*	*Proteus vulgaris*
**Control**	0	0	0	0	0
**15 mg/mL**	24.4 ± 0.12 ***	22 ± 0.07 ***	20 ± 0.16 ***	17 ± 0.3 ***	NZ ^ns^

Significantly different from control at *** *p* < 0.001, non-significantly different from control at ^ns^ *p* > 0.05. Note: N.Z—No zone of inhibition; the values are mean ± standard deviation (*n* = 3).
